# Prediction Models of Shear Parameters and Dynamic Creep Instability for Asphalt Mixture under Different High Temperatures

**DOI:** 10.3390/polym13152542

**Published:** 2021-07-31

**Authors:** Junxiu Lv, Xiaoyuan Zhang

**Affiliations:** 1 The Architectural Design & Research Institute of Zhejiang University Co., Ltd., Hangzhou 310028, China; lvjunxiu1112@163.com; 2 School of Transportation, Southeast University, Nanjing 210096, China; 3 School of Civil Engineering and Architecture, Zhejiang Sci-Tech University, Hangzhou 310018, China

**Keywords:** asphalt mixture, shear strength, high-temperature creep, stress level, triaxial test

## Abstract

This study mainly investigates the prediction models of shear parameters and dynamic creep instability for asphalt mixture under different high temperatures to reveal the instability mechanism of the rutting for asphalt pavement. Cohesive force *c* and internal friction angle *φ* in the shear strength parameters for asphalt mixture were obtained by the triaxial compressive strength test. Then, through analyzing the influence of different temperatures on parameters *c* and *φ*, the prediction models of shear strength parameters related to temperature were developed. Meanwhile, the corresponding forecast model related to confining pressure and shear strength parameters was obtained by simplifying the calculation method of shear stress level on the failure surface under cyclic loading. Thus, the relationship of shear stress level with temperature was established. Furthermore, the cyclic time *F_N_* of dynamic creep instability at 60 °C was obtained by the triaxial dynamic creep test, and the effects of confining pressure and shear stress level were considered. Results showed that *F_N_* decreases exponentially with the increase in stress levels under the same confining pressure and increases with the increase in confining pressure. The ratio between shear stress level and corresponding shear strength under the same confining pressure was introduced; thus, the relationship curve of *F_N_* with shear stress level can eliminate the effect of different confining pressures. The instability prediction model of *F_N_* for asphalt mixture was established using exponential model fitting analysis, and the rationality of the model was verified. Finally, the change rule of the parameters in the instability prediction model was investigated by further changing the temperature, and the instability forecast model in the range of high temperature for the same gradation mixture was established by the interpolation calculation.

## 1. Introduction

As a comprehensive effect of traffic volume increase, channelized traffic, and sustained high-temperature weather, rutting has become one of the main pavement diseases, and the asphalt pavement with semi-rigid base is generally adopted in China [1,2]. Thus, rutting occurs mainly in the asphalt layer because of the high strength from the semi-rigid base. The usage perspective of the existing highway in Jiangsu province of China indicates that the rutting instability of asphalt pavement will occur in six to 10 years, where the instability time is related to the mixture type, load level, temperature, pavement structure, and other factors. If the deformation law of asphalt pavement is obtained prior to the occurrence of rutting instability, then the instability time can be predicted. Therefore, improving the mixture performance indicators is greatly important in determining the reasonable preventive maintenance time and extending the rutting instability time of asphalt pavement.

### 1.1. Test Methods of High-Temperature Deformation

Lack of high-temperature stability for asphalt mixture is the fundamental cause of the rutting disease, which seriously influences the quality of asphalt pavement. Some researchers at home and abroad perfected the studies and evaluation methods for high-temperature deformation of asphalt pavement for the rutting stability.

For example, the researchers measured the indicators about stability and flow values through Marshall tests to determine the high-temperature stability of asphalt mixture. The method has been widely used for a long time because the entire test operation is simple [2]. However, these indicators cannot explain the reason for permanent deformation, which is only a summary of the empirical judgment method. Furthermore, some researchers put forward corresponding rutting tests about the environment and stress state more consistently with the actual situation to supplement the method of stability evaluation for asphalt mixture, such as the laboratory rutting test, large ring, and straight test. However, the stress state of the mixture is relatively complex in the rutting test process, and the actual deformation value cannot be used in the fitting analysis of mechanical parameters. As a result, the established forecast model of pavement rutting is only an empirical model; thus, it is limited and difficult to apply [3]. Peng et al. [4] explored the effect of car speed, accumulated deformation, and maximum permanent deformation on the high-temperature performance of asphalt mixture to obtain more complete evaluation index, and the Wheel Rutting Index (WRI), which can better reflect the development law of rutting and distinguish the stable performance, was put forward.

The asphalt pavement rutting is the result of permanent deformation accumulation for asphalt mixture under the repeated traffic load [5,6]. Researchers conducted creep tests based on mechanics principle to further study the deformation law of asphalt mixture [7,8,9]. Fan et al. [7] analyzed the effect of gradation, oil–stone ratio, temperature, and loads on the creep stiffness modulus for asphalt mixture by the static and dynamic creep tests. The results showed that two types of tests are in the same change tendency and have good correlation, but the temperature sensitivity of dynamic creep stiffness modulus is relatively higher. Goh et al. [8] proposed a simple approach to determine the flow cycle times of asphalt mixture under the dynamic creep test. The flow cycle times were defined as the minimum point of strain rate versus loading cycle times by the modified data point. Zhang et al. [9] carried out the dynamic triaxial creep test for AC20 gradation asphalt mixture and analyzed the effect of environment temperature and axial loads on the deformation characteristics of the mixture. Thus, they pointed out that the rheological cyclic times at the mixture instability or the minimum value of the permanent strain rate with the load cycles times can serve as an evaluation index of high-temperature stability. The specimen of the triaxial creep test is closer to the practical working situation and the test results are relatively more reasonable than those of the uniaxial creep tests.

### 1.2. Instability Mechanism of High-Temperature Deformation

The two types of mechanism of producing permanent deformation for asphalt mixture are deformation of volume change under pressure loads and deformation of volume constant under shear loads. Bekheet [8] showed that accumulation of pavement rutting deformation mainly comes from the shear flow of asphalt pavement materials because the base strength of the actual pavement is large. The shortage of the high-temperature stability for asphalt mixture is the key reason of the rutting instability, which can be traced to a low-specific material strength or the large cumulative plastic deformation [10]. When the shear stress of the pavement mixture increases and the shear strength of the mixture is more under repeated vehicle loading, accumulated deformation eventually leads to the rutting instability.

Researchers at home and abroad have fully realized that the shortage of shear strength for asphalt mixture is an important reason for rutting disease in actual pavements, and the performance of mixture has been investigated by shear strength tests [11,12,13]. Peng et al. [11] analyzed the effect of material parameters, such as void fraction, on the performance of the overall shear strength for asphalt mixture through the uniaxial penetration test with unconfined compressive strength test. Gao et al. [12] put forward a penetration test model by using finite element simulation analysis on the basis of the elastic layer theory, similar to actual pavement stress. Then, the failure envelope of the mixture was obtained by analyzing the results of the penetration test and the unconfined compressive strength test, thereby obtaining the values of shear strength parameters for cohesive force *c* and internal friction angle *φ*. Furthermore, some scholars have conducted studies on the high-temperature deformation law based on the shear strength of asphalt mixture.

However, in the practical usage process of asphalt pavement, the instability of rutting is usually not the result of vehicle loads at one cyclic; it is eventually the damage failure from a long-term repeated loading [13,14]. For instance, some researchers have conducted an in-depth study on the dynamic shear creep properties of asphalt mixture. Fwa et al. [15] proposed a rutting depth prediction model based on the consideration of shear deformation and bearing capacity of asphalt mixtures. The predicted results of the model were demonstrated by the laboratory wheel tracking test. Yuan et al. [16] explored the effect of temperature on the dynamic shear deformation of asphalt mixture and found that shear deformation increased in the heating and cooling process. Zhang et al. [17] conducted decomposition of the total strain for asphalt mixture under repeated loads, including elastic, viscoelastic, plastic, viscoplastic strain, and viscous fracture strain. Fontes et al. [18] studied the permanent deformation performance of asphalt rubber mixtures by the repeated simple shear test at constant height and the wheel tracking test. The testing results showed that the asphalt rubber mixtures can significantly enhance the resistance to rutting.

The essence of the creep deformation accumulated instability for asphalt mixture at high temperature is the result of the material subjected to shear stress over and over again. Therefore, from the instability failure perspective of cyclic pressure and shear for asphalt pavement under different load actions, the study on the effect of creep instability of asphalt mixture on the pavement rutting failure is feasible. However, these studies about the instability of mixture mainly focus on the degree of deformation but neglect the occurrence time of instability, which is important to conduct timely maintenance and management of asphalt pavement.

## 2. Objective

The present study mainly considers variables, such as temperature and load conditions, to analyze the creep instability characteristics of asphalt mixture by triaxial tests and put forward the instability prediction model of asphalt mixture at the entire temperature range. This research process can provide a method to determine the maintenance time at actual pavement. Specific contents are as follows:

(1) To establish the prediction model of shear strength parameters, triaxial compressive strength tests (TCST) that measure strength of asphalt mixture under different confining pressures are used to obtain shear strength parameters for a given temperature based on the Mohr–Coulomb strength theory. Then, the influence of different temperatures is considered to identify the relationship between shear strength parameters of asphalt mixture and temperature. Meanwhile, the influence of the additive on shear strength parameters of asphalt mixture is explored. Furthermore, the prediction model of shear stress level in the failure surface under cyclic loading is developed through geometric analysis.
(2) The effects of the confining pressure and compressive load level on the test results are considered to establish the instability prediction model under triaxial dynamic creep tests (TDCT), and the prediction model of the dynamic creep cycle times is developed by fitting analysis at 60 °C. Moreover, the influence of temperature on the shear strength is comprehensively considered to obtain the predicted equation of cyclic times for the mixture in the entire temperature range and to build and verify the instability prediction model in the laboratory test.

## 3. Raw Materials and Testing Methods

### 3.1. Raw Materials

**Figure 1:**
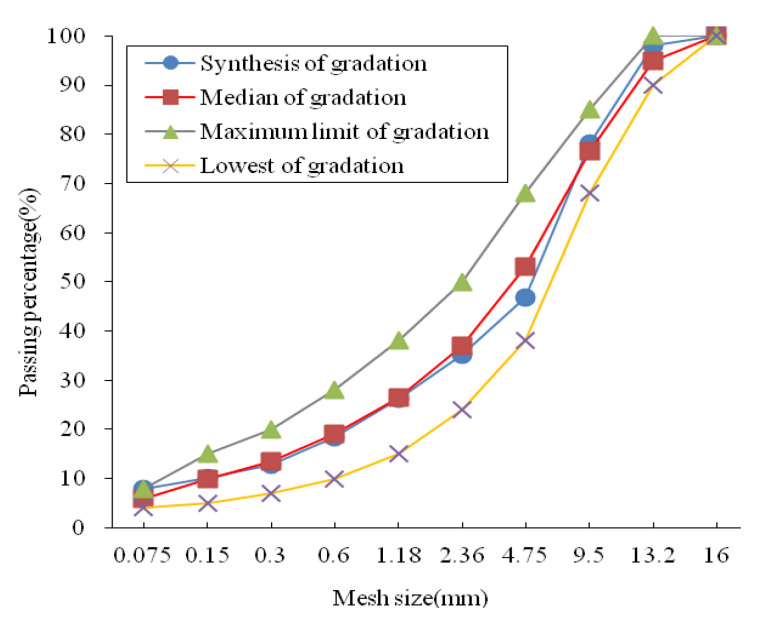
Gradation curve of AC13 asphalt mixture.

Typical AC13 dense-graded asphalt mixture is selected as the research object, and the correlated gradation curve is shown in Figure 1. Asphalt is adopted as the SBS modified bitumen, and coarse and fine aggregates are selected as the basalt and limestone stones, respectively; mineral powder is unified as limestone (Zhejiang Transportation Resources Investment Co., Ltd., Hangzhou, China). According to the test method of [19], testing indexes of the basic performance were measured, and all results satisfied the technical specification for construction of the highway asphalt pavement [20]. In addition, the additive is selected as the basalt short fiber, and the fiber performance and basic parameters are shown in the literature [21].

The target voidage of asphalt mixture is set to 4%, and the optimal oil–stone ratio for asphalt mixture is 4.9% by using the Superpave design method.

### 3.2. Testing Methods

#### 3.2.1. Specimen Preparation and Loading

The result in the triaxial test is closer to the actual situation of asphalt pavement than the stress state of the specimen in the uniaxial test. Furthermore, the influence of confining pressure in the triaxial test on the performance parameters and other testing results for asphalt mixture can be studied by changing the laboratory gas pressure. For instance, the shear strength parameters of asphalt mixture can be obtained by the TCST. In addition, the TDCT was carried out to obtain the high-temperature instability life of asphalt mixture under a confining pressure and cyclic vertical pressure.

**Figure 2:**
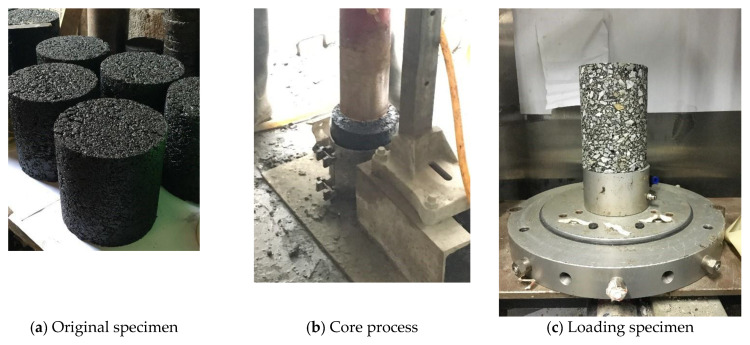
Process of specimen forming. (**a**) original specimen, (**b**) Core process, (**c**) Loading specimen.

For the specimen formation of triaxial tests, the cylindrical specimen with a diameter of 150 mm and a height of 170 mm was formed by a rotary compactor (Pine Instrument Company, Grove City, PA, USA), and then the core specimen with a diameter of 100 mm and a height of 150 mm was obtained by drilling the cylindrical specimen. The specimen formation process is shown in Figure 2. Three parallel specimens were selected under the same testing condition.

**Figure 3:**
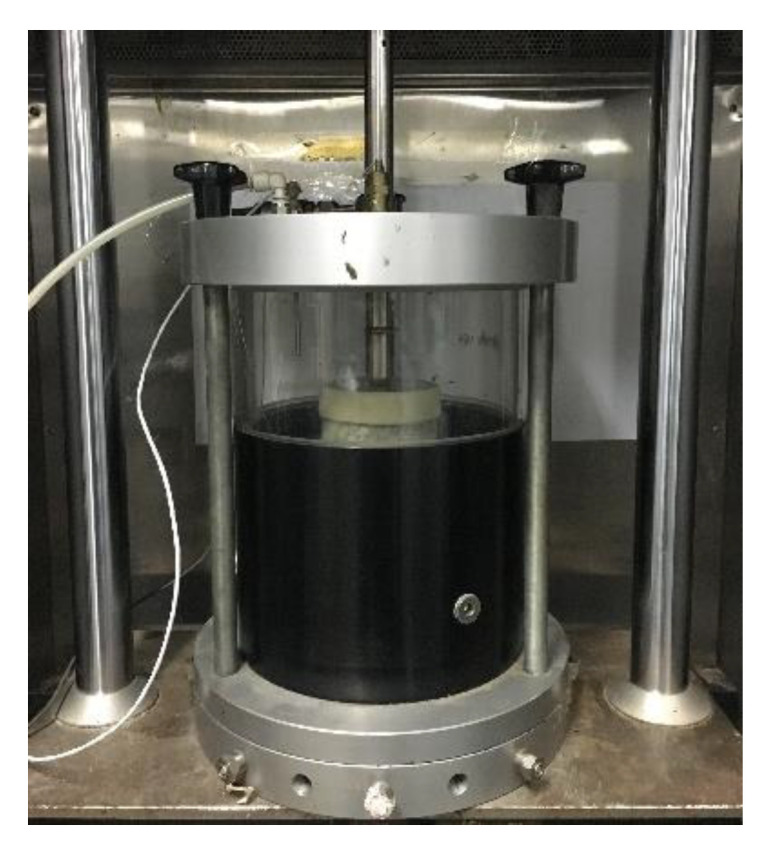
Equipment of triaxial tests.

All the triaxial tests were carried out in the UTM-25 universal testing machine (IPC Global, Melbourne, Australia), as shown in Figure 3. The specimens must be stored in a stable temperature for 4 h in UTM-25 prior to testing to ensure that the internal temperature of the testing specimens can reach the set loading condition. Meanwhile, the semi-sine wave load in the TDCT is selected, where the process is loading of 0.1 s and the unloading of 0.9 s [8,22].

#### 3.2.2. Scheme of the TCST

**Figure 4:**
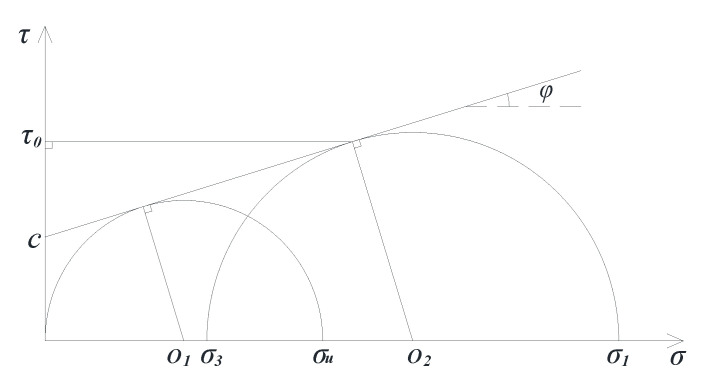
Schematic of the solution for *c*, *φ*, and *τ*_0_.

TCST can simulate the simple stress state of a point in the materials. The corresponding peak value of the vertical stress under the different confining pressures can be obtained by changing the gas pressure in the triaxial chamber. Then, according to the Mohr–Coulomb theory, a series of Mohr circles and corresponding tangent lines, considered the failure envelope, can be obtained. Furthermore, the values of cohesive force *c*, internal friction angle *φ*, and shear stress strength *τ*_0_ on the failure surface can be calculated. Specifically, the compressive strength *σ_u_* and *σ*_1_ corresponding to zero confining pressure and non-zero confining pressure *σ*_3_ are obtained at the same temperature, and then Mohr semicircles (shown in Figure 4) are designed to obtain the parameters *c*, *φ*, and *τ*_0_, the calculation formula of which are as follows:

(1) $$\varphi =\arcsin(\frac{\sigma_{1}-\sigma_{3}-\sigma_{u}}{\sigma_{1}+\sigma_{3}-\sigma_{u}})$$

(2) $$c=\frac{\sigma_{u}}{2}(\frac{1-\sin\varphi }{\cos\varphi })$$

(3) $$\tau_{0}=\frac{\sigma_{1}-\sigma_{3}}{2}\cos\varphi$$

For temperature setting of specimens, when the maximum environment temperature reaches 40 °C in summer, the mixture temperature is highest in the area of 2 cm depth from the asphalt pavement surface, and it can almost increase to 60 °C. A series of high-temperature values was selected to approach the actual road surface temperature to study the temperature effect on the shear parameters of AC13 asphalt mixture as the upper layer material in asphalt pavement. The specific test scheme is as follows:

(a) The specimens were set to 70 °C, 60 °C, 50 °C, and 40 °C in the UTM-25 testing machine, respectively, and the shear strength parameters under three different confining pressures were obtained at each same temperature.
(b) The relationship between shear strength parameters and high temperature was established and analyzed. Then, the change rule of shear strength parameters in asphalt mixture after adding fiber additives at a specific temperature was investigated.

#### 3.2.3. Scheme of the TDCT

**Figure 5:**
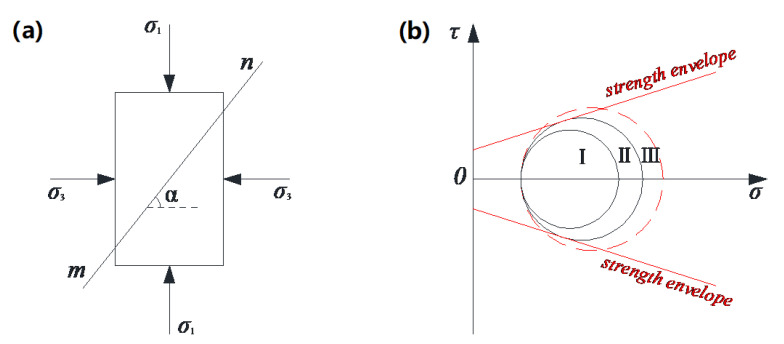
Schematic of Mohr–Coulomb failure criterion: (**a**) Failure surface (**b**) Envelope lines.

According to the Mohr–Coulomb failure criterion, when the ratio of shear stress to normal stress on a stress plane reaches a certain value, the soil undergoes shear failure along the stress plane, and the location of the failure plane is only related to the shear parameters *c*, *φ* of the material. TCST can be used to obtain the compressive strength values under different confining pressures, thereby determining the failure envelope of the material, as shown in Figure 5. Similar to the soil particles, asphalt mixture also has evident granular characteristics, and its strength consists of the cohesive force provided by asphalt and the internal friction force between aggregates [23,24,25].

The internal stress state of the actual asphalt pavement under vehicle loads is similar to that of the triaxial dynamic creep specimen with confining pressure. Moreover, the internal stress of asphalt mixture under most loads is shown in area I of Figure 5b, that is, the Mohr circle of equivalent stress is all at the inside of the failure envelope lines.

Research showed that the creep process of asphalt mixture can be divided into three stages, as follows [5,8,26]:

(1) initial compaction stage, in this stage the internal structure of the mixture is eadjusted, and the strain gradually increases and the corresponding strain rate decreases;
(2) in the stable growth stage, the viscoplastic deformation of the mixture is accumulated continuously, the internal aggregate begins to break, the strain increases steadily, and the strain rate remains unchanged;
(3) in the accelerated instability stage, the instability of the mixture structure occurs, and the strain and the strain rate increases rapidly.

The starting point of the creep third stage can be regarded as the point with the minimum strain rate in the process, and the corresponding cyclic loading times *F_N_* can be used as an important index to evaluate the high-temperature stability of the asphalt mixture. The asphalt mixture suffers irreversible damage under the action of cyclic loads, and then instability on the surface of the maximum shear stress occurs. Therefore, the deformation and instability of the mixture under different loads can be studied from the perspective of cyclic compression and shear failure. The specific test scheme is as follows:

(a) TDCT was conducted by setting different confining pressures, including 0, 69, and 138 kPa at a high temperature of 60 °C to obtain the curve changes with confining pressures as the only variable, and then establish an instability prediction model.
(b) TDCT was carried out on asphalt mixture at different temperatures to obtain the change in the instability prediction model with temperature as the only variable.

Therefore, temperature and confining pressures are the variables of the testing process. We strive to find the instability prediction model of asphalt mixture within the full temperature range through the test and analysis.

## 4. Discuss and Analysis

### 4.1. Influence Factors and Prediction Model of Shear Parameters in Asphalt Mixture

#### 4.1.1. Effect of Temperature on Shear Strength Parameters

**Figure 6:**
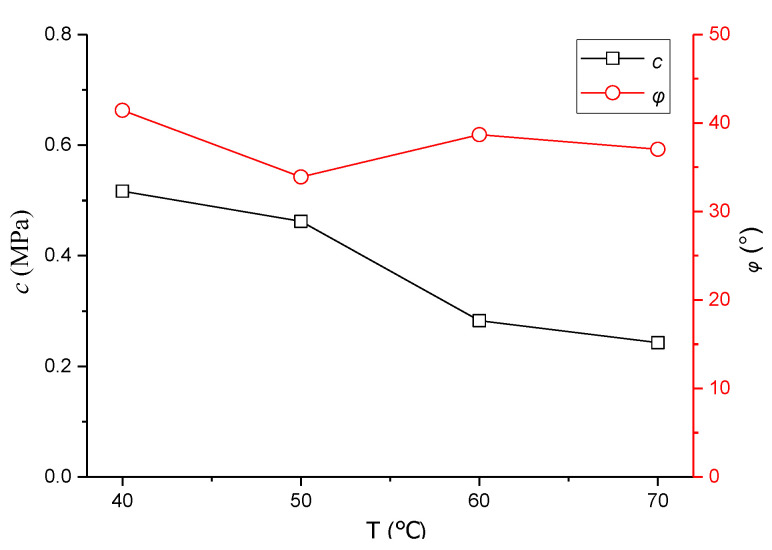
Effect of temperature on shear parameters.

Through TCSTs under different temperatures, the compressive strength values under different confining pressures are exhibited in Table 1, and the results of shear parameters *c*, *φ* with temperature are shown in Figure 6.

Figure 6 shows that the value of *c* decreases with the increase in temperature. When the temperature rises from 50 °C to 60 °C, the decrease in *c* is the most evident, namely, it is the most sensitive to temperature. Meanwhile, the result of *φ* fluctuates with the change in temperature. When temperature increases from 40 °C to 50 °C, the decrease in *φ* is the greatest, and it is the most sensitive to temperature. When the temperature continues to increase from 50 °C, *φ* presents a fluctuating trend, and it increases slightly to 60 °C. This finding is caused by the large decline in *c* value at this time, and asphalt mixture easily obtains small dislocation due to the lack of cohesion under the stress state; thus, the effect of the friction angle can be fully reflected. This finding also explains that the measured *φ* of asphalt mixture at 60 °C and 70 °C is slightly greater than that at 50 °C.

#### 4.1.2. Effect of Fiber on Shear Strength Parameters

**Figure 7:**
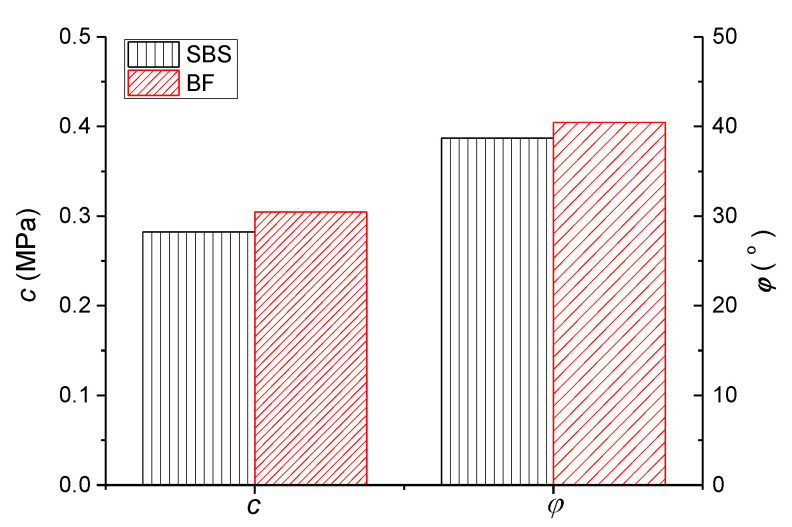
Comparison results of the shear parameters with and without fiber mixture.

Combined with the engineering practice, to improve the high-temperature stability of the pavement mixture, asphalt mixture is evaluated by adding a certain content fiber [21,27,28]. Basalt fiber of 0.3% mixture weight was selected to add into the mixture to conduct the relevant studies, and results of the TCST at 60 °C after adding fiber are shown in Table 2. Meanwhile, the comparisons of shear strength parameters with and without fiber asphalt mixture are shown in Figure 7, where SBS represents the control specimen without fiber, and BF represents the specimen with basalt fiber addition.

Figure 7 shows that, compared with the shear parameters *c*, *φ* of the control specimen, the results after adding basalt fiber are increased by 7.9% and 4.6%, respectively. This finding indicates that after adding fiber to the mixture, the adhesive property of asphalt mortar is improved; thus, *c* is increased. In addition, when the aggregate moves under stress, overcoming the viscous resistance of the asphalt film wrapped on the surface of aggregates, as well as the friction resistance between aggregates is necessary. Thus, the addition of fiber in the mixture also increases the *φ* value to some extent.

#### 4.1.3. Establishment of Prediction Models of Shear Parameters

(i) Prediction Models of *c* and *φ*

**Figure 8:**
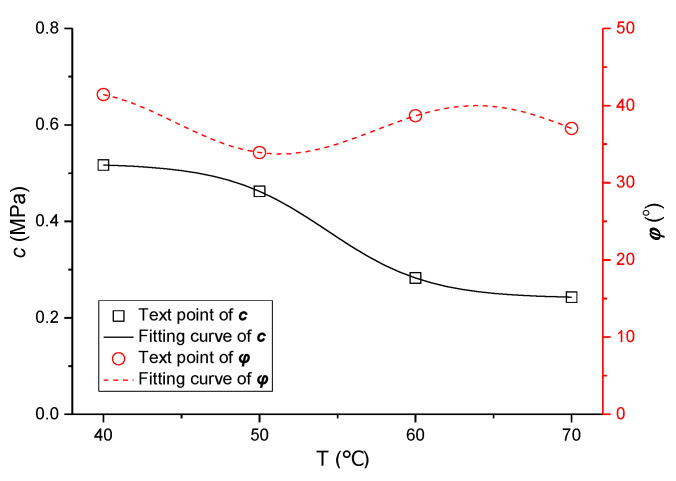
Fitting curve of shear parameters *c* and *φ*.

Combined with the variation of shear strength parameters in Figure 6, the following equations were selected to fit the values of *c* and *φ*:

(4) $$y=b_{1}+\frac{a_{1}{+b}_{1}}{1+\exp(\frac{x-c_{1}}{d_{1}})}$$

(5) $$y=a_{2}x^{b_{2}}+c_{2}\sin x^{d_{2}}$$

where a_1_, b_1_, c_1_, and d_1_ are the parameters of Equation (4) to be solved, and the relationship between cohesive force *c* and temperature *T* for the mixture is established; a_2_, b_2_, c_2_, and d_2_ are the parameters of Equation (5) to be solved, and the relationship between internal friction angle *φ* and *T* is established. The fitting curve is shown in Figure 8.

Thus, the prediction model of the cohesive force *c* for asphalt mixture can be obtained as follows:

(6) $$c=b_{1}+\frac{a_{1}{+b}_{1}}{1+\exp(\frac{T-c_{1}}{d_{1}})}$$

where a_1_ = 0.5201, b_1_ = 0.2406, c_1_ = 54.36, d_1_ = 3.246, and the correlation index *R*^2^ is 0.9912.

Meanwhile, the prediction model of the internal friction angle *φ* for asphalt mixture is obtained as follows:

(7) $$\varphi =a_{2}T^{b_{2}}+c_{2}\sin T^{d_{2}}$$

where a_2_ = 53.89, b_2_ = −0.0939, c_2_ = 3.536, d_2_ = 0.7248, and the correlation index *R*^2^ is 0.9851.

(ii) Prediction Model of Shear Stress on the Failure Surface

**Figure 9:**
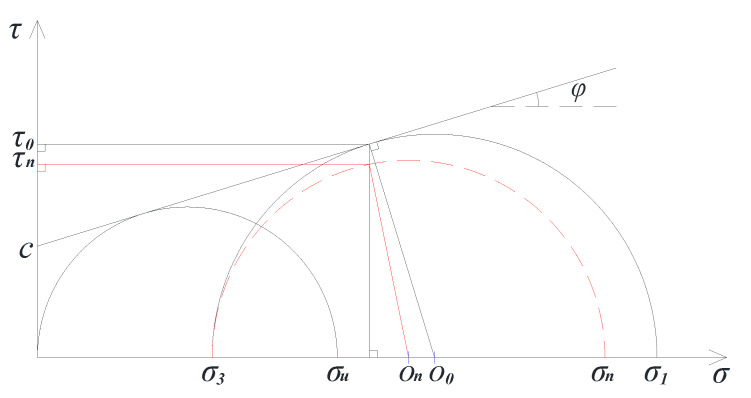
Schematic for calculating shear stress on failure surface.

Furthermore, the prediction model of shear stress *τ* on the failure surface is solved using the Mohr–Coulomb theory to obtain a function of *c* and *φ*. The solution schematic and calculation method are as follows (Figure 9):

Figure 9 shows that *τ*_0_ is the shear stress strength on the failure surface of the asphalt mixture when it is an instable failure by one-time compression, and it can be calculated by Equation (3). Furthermore, by using Equations (1)–(3) to eliminate intermediate variables, the prediction model of shear stress strength *τ*_0_ on the failure surface of asphalt mixture under one-time compression only contains *c*, *φ*, and *σ*_3_, as follows:

(8) $$\tau_{0}=\frac{\sigma_{3}\sin\varphi +c\cos\varphi }{1-\sin\varphi }\cos\varphi$$

Meanwhile, assuming the failure surface of the specimen does not change under cyclic loading, the corresponding shear stress *τ_n_* on the failure surface can be calculated by the following equation:

(9) $$\tau_{n}=\sqrt{{\left(\frac{\sigma_{n}-\sigma_{3}}{2}\right)}^{2}-{\left(\frac{\sigma_{1}-\sigma_{3}}{2}\sin\varphi -\frac{\sigma_{1}-\sigma_{n}}{2}\right)}^{2}}$$

where *σ_n_* is the vertical pressure stress level under cyclic loading.

Equations (1), (2) and (9) are used to eliminate intermediate variables, and the prediction model of shear stress level *τ_n_* on the failure surface of asphalt mixture under cyclic loading only contains *c*, *φ*, *σ*_3_, and *σ_n_* is obtained as follows:

(10) $$\tau_{n}=\sqrt{\left(\sigma_{3}\sin\varphi +c\cos\varphi )[\sigma_{n}-(1+\sin\varphi )\sigma_{3}-c\cos\varphi \right]}$$

Equations (6)–(8) and (10) are combined, and the quantitative relationship between the prediction model of shear stress on the failure surface and temperature can obtain the stress state.

### 4.2. Creep Instability Life and Its Prediction Model of Asphalt Mixture

#### 4.2.1. TDCT Results of Asphalt Mixture at 60 °C

**Figure 10:**
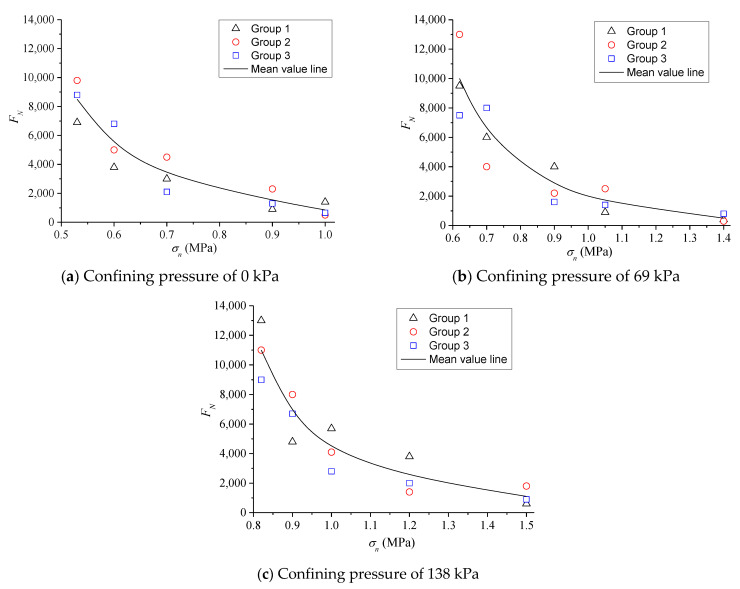
*F_N_* under different pressure stress level and confining pressure (**a**–**c**) at 60 °C.

The TCST results indicate that the influence of cyclic loads of less than the compressive strength value is considered to conduct the TDCT. Three parallel tests were selected under each load level, and the test results at a high temperature of 60 °C are shown in Figure 10.

**Figure 11:**
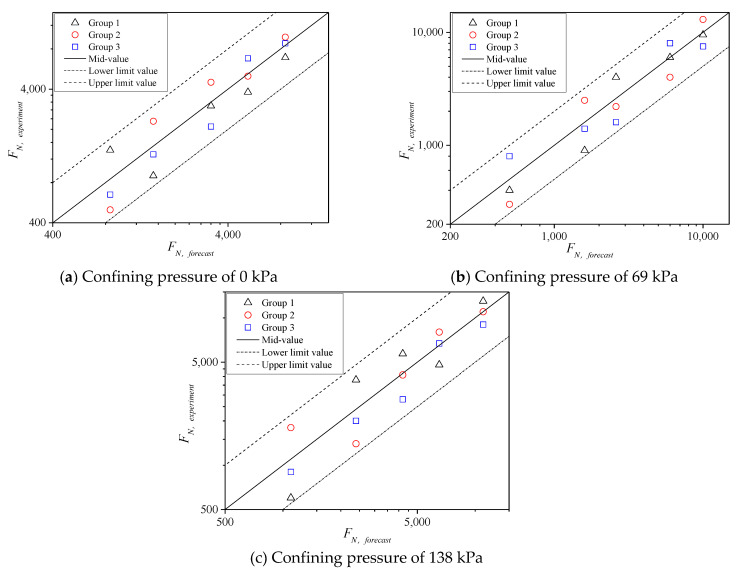
Validity of *F_N_* under different confining pressure at 60 °C.

According to the results of Figure 10, the cyclic loading times *F_N_* of creep instability decreases exponentially with the increase in the vertical pressure stress levels *σ_n_* under the same confining pressure. In addition, *F_N_* increases gradually with the increase in confining pressure at the same *σ_n_*. Furthermore, validity analysis was conducted on the results of three groups of parallel tests under various confining pressures, i.e., the test results of *F_N_* corresponding to 15 specimens. The instability failure points fall in the dispersion band of twice the safety factors of the testing mean, as shown in Figure 11. Therefore, the results showed that *F_N_* values obtained from the testing are reasonable [26]. The *σ_n_* of specimens subjected to the cyclic load is larger, the specimen is earlier closed to the failure state, and the dispersion of datum is relatively larger. As *σ_n_* gradually decreases, *F_N_* values are closer to the median line, that is, the dispersion is also smaller. Generally, it is more reasonable for subsequent analysis.

#### 4.2.2. Instability Prediction Model and its Verification of Asphalt Mixture at 60 °C

(i) Presentation of the Instability Prediction Model

**Figure 12:**
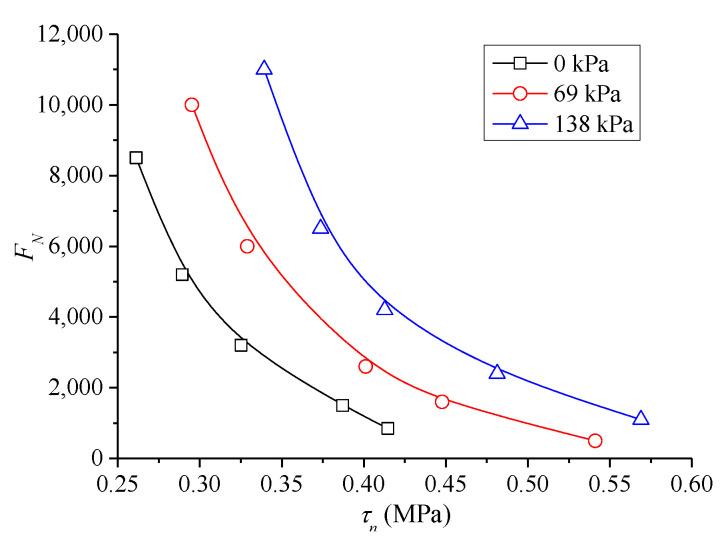
Curve chart of *F_N_* with shear stress level.

For the results of the TDCT at 60 °C, the mean value of *F_N_* was considered the creep life of asphalt mixture. Furthermore, according to formula (10), the relationship between shear stress level *τ_n_* and creep instability of the mixture was investigated from the perspective of cyclic compression and shear failure, and the results are shown in Figure 12. The figure shows that, for the mixture under the same confining pressure, *F_N_* increases exponentially with the decrease in *τ_n_*, and it tends to infinity when *τ_n_* decreases to a certain extent. Thus, the entire process has an evident fatigue trend phenomenologically. In addition, the larger confining pressure indicates longer instability life and larger life limit of the mixture.

**Figure 13:**
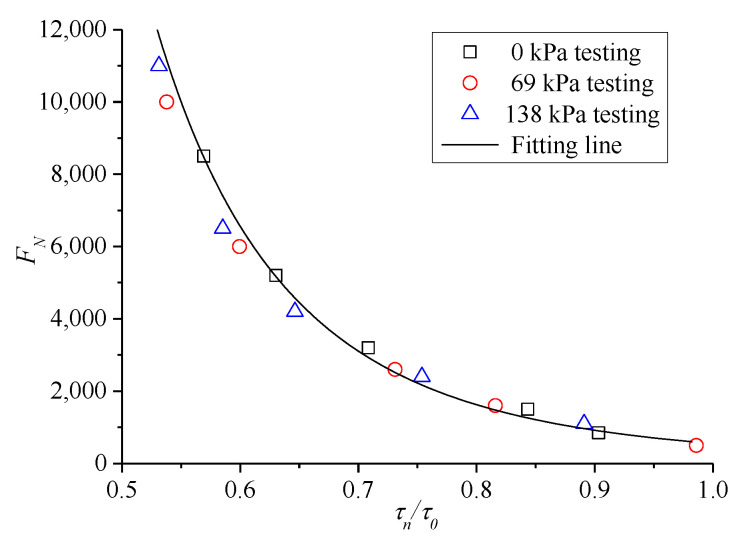
Curve chart of *F_N_* with shear stress ratio.

The ratio between *τ_n_* and corresponding shear strength *τ*_0_ under the same confining pressure was introduced, namely, *τ_n_*/*τ*_0_ is the X-axis of the relationship curve to obtain a relatively uniform instability prediction model for asphalt mixture, and the corresponding *F_N_* is still the Y-axis. Therefore, the resulting curve can eliminate the effect of different confining pressures, and the results are shown in Figure 13.

The following exponential function model was selected to fit the results in Figure 13, as follows:

(11) $$F_{N}=a{\left(\frac{\tau_{n}}{\tau_{0}}\right)}^{b}$$

where a and b are the parameters to be solved in the model. The fitted results are a = 550.5, b = −4.851, and the correlation index *R*^2^ is 0.9713.

In the prediction model (11), *τ_n_* and *τ*_0_ can be substituted by the formulas (10) and (8), respectively. Therefore, when the gradation of the mixture is unchanged, only the values of *c* and *φ* in the prediction model are measured, and a reasonable cyclic stress level *σ_n_* under a certain confining pressure *σ*_3_ is given. Then, the shear stress ratio *τ_n_*/*τ*_0_ in the prediction model can be obtained using formulas (10) and (8). Finally, the *F_N_* of creep instability under the corresponding stress state can be obtained by prediction model (11).

(ii) Verification of the instability prediction model

Combined with these rules, adding fibers can change the properties of binder in asphalt mixture, and thus increase the shear strength parameters. A certain content of basalt fiber was selected to add into the asphalt mixture, and then *F_N_* of the fiber asphalt mixture was predicted by the prediction model (11) to verify the relevant tests.

Specifically, the shear parameter values of adding 0.3% basalt fiber asphalt mixture were obtained by the TCST at 60 °C, and the results are shown in Table 2. Then, under the confining pressure of 138 kPa, TDCTs were conducted on the fiber asphalt mixture at stress levels *σ_n_* of 0.9, 1.0, 1.2, and 1.5 MPa, where *σ_n_* was lower than the compressive strength *σ*_0_ (i.e., 1.969 MPa). Furthermore, through the stress transformation, the results of shear stress strength, shear stress level, shear stress ratio, and *F_N_* under cyclic loading are shown in Table 3.

The comparison of Table 3 and Figure 10c shows that under the confining pressure of 138 kPa, the mean values of *F_N_* for fiber asphalt mixture at the same stress level increase significantly, that is, the occurrence time of creep instability is prolonged. Thus, adding fiber improves the high-temperature stability of asphalt pavement.

**Figure 14:**
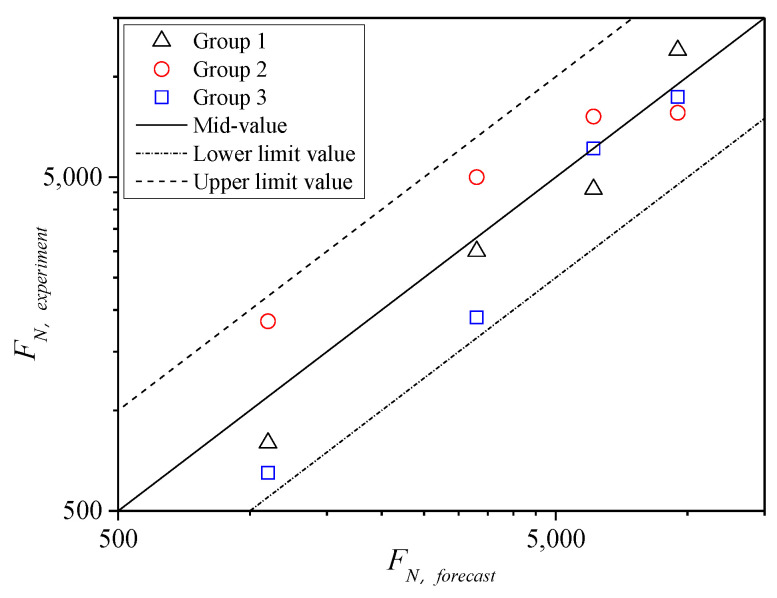
Distribution of *F_N_* for fiber asphalt mixture.

The analysis of the validity of 12 *F_N_* values for fiber asphalt mixture indicates that the test results of *F_N_* fall within the dispersion band of twice safety factors (Figure 14). It also indicates that the *F_N_* values obtained from the TDCT are reasonable and can be used for subsequent analysis.

**Figure 15:**
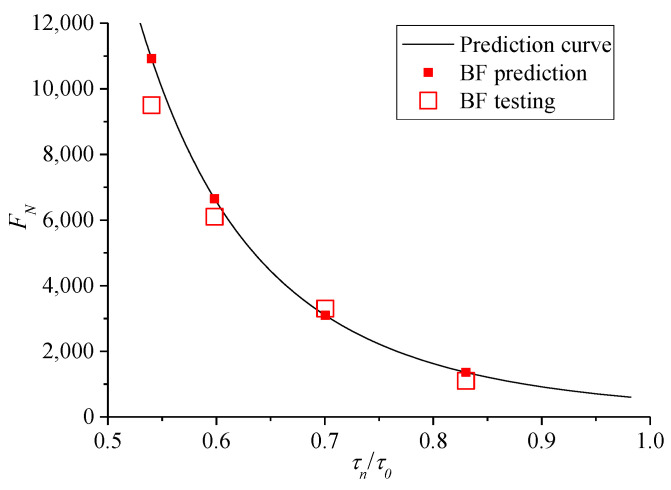
Predicted and experimental *F_N_* values for fiber asphalt mixture.

The mean values of the test results of *F_N_*, namely, the median values of the dispersion band of twice safety factor, which was considered the instability life of the specimen. Moreover, the predicted *F_N_* of the fiber asphalt mixture under the same stress state are shown in Figure 15. The figure shows that when the shear stress ratio *τ_n_*/*τ*_0_ of the fiber asphalt mixture is the same, the predicted *F_N_* deviate from that of the TDCT to some degree. However, the tested *F_N_* is evenly distributed on both sides of the predicted curve; thus, the prediction model can be verified.

#### 4.2.3. Instability Prediction Model of Asphalt Mixture in the Entire Temperature Range

(i) Presentation of the Prediction Model

**Figure 16:**
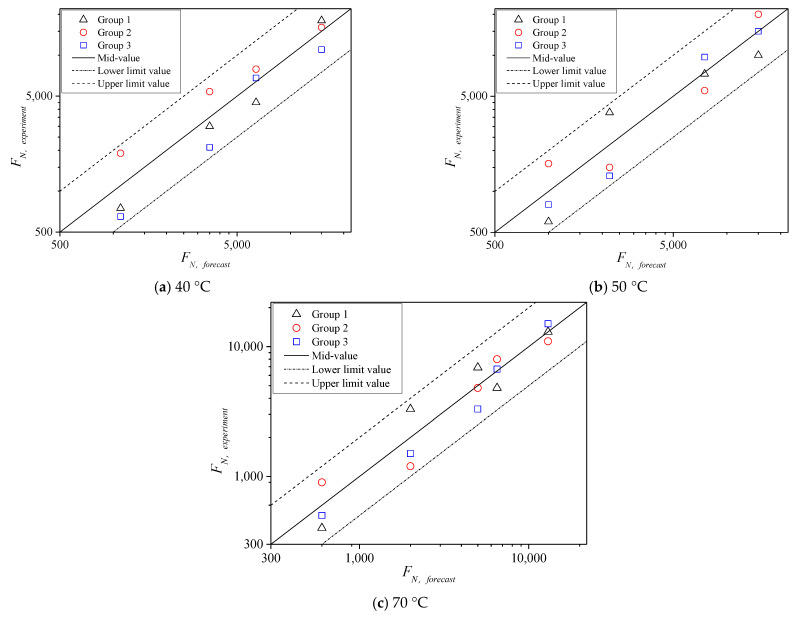
Distribution of *F_N_* at different temperatures.

On the basis of shear parameters obtained from the TCST at different temperatures and the corresponding confining pressures, temperatures of 40 °C, 50 °C, and 70 °C were further considered to conduct the TDCT by setting compressive stress levels *σ_n_* less than the corresponding compressive strength under the 0 kPa confining pressure (Table 1), where *σ_n_* was set to 1.25, 1.4, 1.5, and 2 MPa for 40 °C; 0.9, 1.05, 1.25, and 1.5 MPa for 50 °C; 0.38, 0.43, 0.48, 0.63, and 0.85 MPa for 70 °C. Meanwhile, three parallel test results under each stress level were analyzed. *F_N_* falls in the dispersion band of twice safety factors of the test mean values, as shown in Figure 16, indicating that the tested *F_N_* values are reasonable.

**Figure 17:**
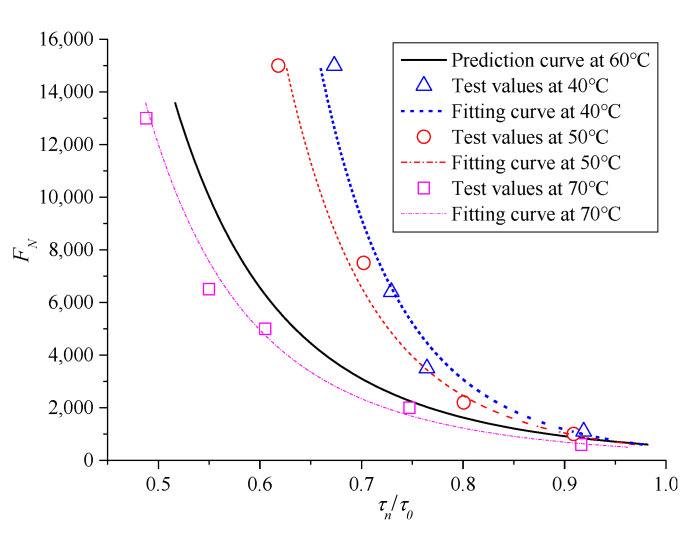
Comparison of *F_N_* values at different temperatures.

**Figure 18:**
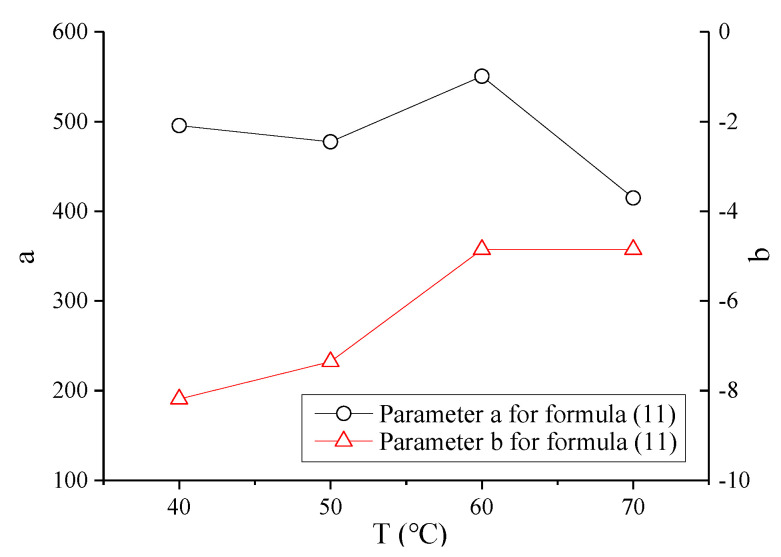
Parameters of the instability prediction model at different temperatures.

Similarly, the mean value of *F_N_* was considered the life of the mixture specimen at various temperatures for further analysis. The test results of *F_N_* with shear stress ratio *τ_n_*/*τ*_0_ on the failure surface of asphalt mixture under zero confining pressure are shown in Figure 17. The prediction model (11) is still used to fit the test results at various temperatures; the fitted deformation curves are represented as dotted lines. Figure 17 shows that the instability life of asphalt mixture and the life limit increase with the decrease in *τ_n_*/*τ*_0_ and temperature, respectively. Therefore, temperature greatly influences the parameters of the mixture in the instability prediction model. The specific values of parameters a, b in the prediction model at various temperatures are fitted by formula (11), and the variation trend with temperature is shown in Figure 18, where the fitted correlation index *R*^2^ is 0.9734, 0.9828, 0.9713, and 0.9737 for 40 °C, 50 °C, 60 °C, and 70 °C, respectively. Within the study temperature range, the prediction model parameters a and b at any temperature were obtained by an interpolation method.

In summary, formula (11) can be considered the basic prediction model at any temperature. When the temperature is known, parameters a, b can be obtained by interpolation, as shown by the results in Figure 18. *τ*_0_ and *τ_n_* can be obtained from formulas (8) and (10), respectively, where *τ*_0_, *τ_n_* are functions related to the confining pressure and the shear strength parameters *c*, *φ*. *c* and *φ* are only related to temperature, and are obtained by the prediction models (6) and (7), respectively. Therefore, the prediction model of *F_N_* for asphalt mixture in the entire temperature range is only related to the temperature, confining pressure, and stress level.

(ii) Verification of the prediction model

**Figure 19:**
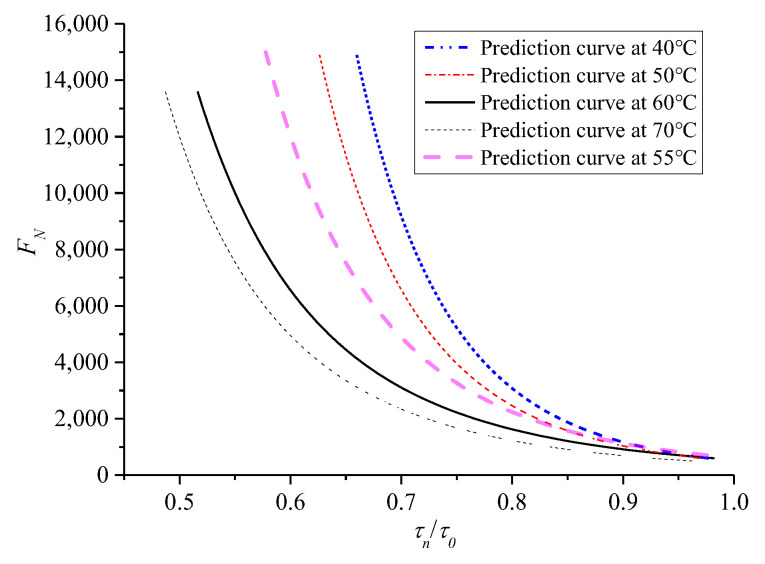
Predicted curves of *F_N_* at different temperatures.

*F_N_* of the mixture specimen at the temperature of 55 °C was evaluated to verify further the validity of the creep instability prediction model for asphalt mixture because this process excludes the test results at this temperature. For the verified process of the prediction model, *T* at 55 °C was substituted into the prediction models (6) and (7), and the corresponding values of *c* and *φ* can be obtained. Then, considering the confining pressure *σ*_3_ at 0 kPa, the corresponding *σ*_3_, *c*, and *φ* were substituted into the prediction models of *τ*_0_ and *τ_n_*, and the corresponding *τ_n_*/*τ*_0_ can be obtained by *σ_n_*. Furthermore, through interpolation for the fitted parameters of the instability prediction model in Figure 18, a and b at 55 °C were obtained as 513.97 and −6.1018, respectively. Finally, the prediction model of *F_N_* can be used to obtain the predicted curve for asphalt mixture at 55 °C, as shown in Figure 19.

**Figure 20:**
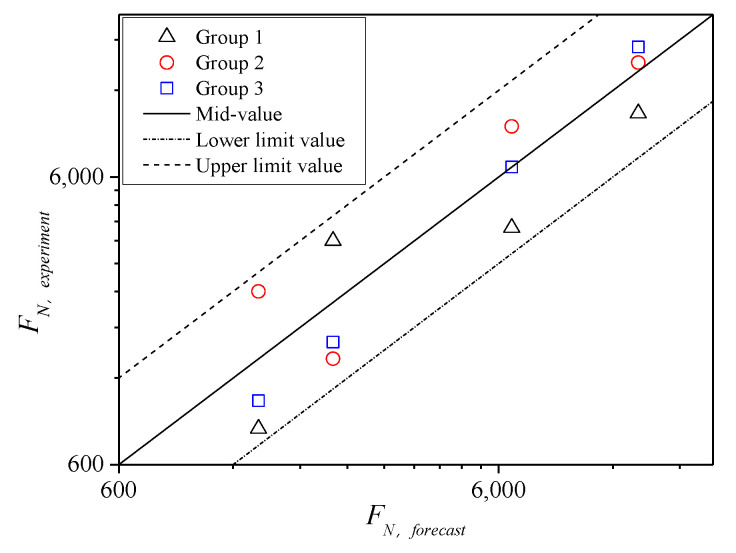
Distribution of tested *F_N_* at 55 °C.

Figure 19 shows that *F_N_* of asphalt mixture increases with the decrease in temperature under the same *τ_n_*/*τ*_0_, and the predicted curve of *F_N_* at 55 °C is in the range of 50 °C to 60 °C. Meanwhile, to verify the predicted *F_N_* at 55 °C, four pressure stress levels were selected as 0.7, 0.85, 1.05, and 1.2 MPa, and three parallel tests were conducted under each stress level. To validate the test results, as shown in Figure 20, the *F_N_* values fall in the dispersion zone of twice safety factors of the tested mean, indicating that the test results are more reasonable and can be used for subsequent analysis.

**Figure 21:**
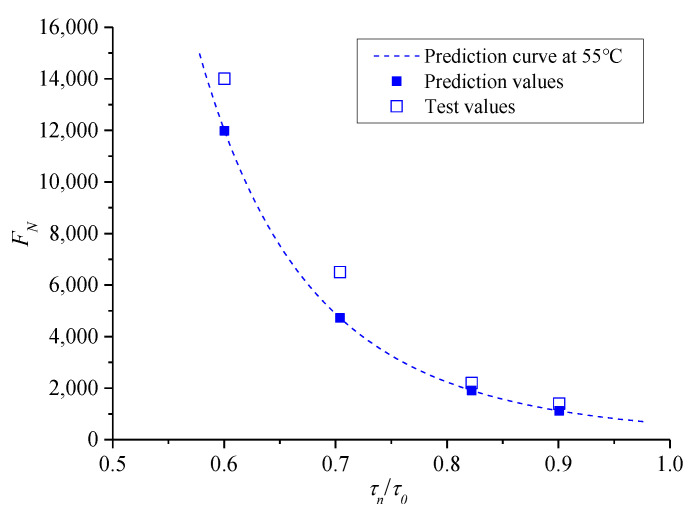
Comparison diagram of predicted and tested *F_N_* at 55 °C.

Furthermore, comparisons between the mean value of the test results and corresponding predicted value under each shear stress ratio are shown in Figure 21. The figure shows that when *τ_n_*/*τ*_0_ is the same, the predicted *F_N_* value is slightly less than the mean value of the test, but this result satisfies the range of the twice safety factor of the experimental mean value. Therefore, the instability prediction model of the asphalt mixture in the entire temperature range is verified. In addition, the result of the prediction model is conservative; thus, it is conducive for timely maintenance and management of asphalt pavement.

## 5. Conclusions

Through the TCST and TDCT, the prediction model of the shear parameters and dynamic creep instability in the entire temperature range for asphalt mixture was investigated. The main conclusions are as follows:

(1) TCSTs show that the cohesive force *c* value of the asphalt mixture decreases with the increase in temperature, and the internal friction angle *φ* value fluctuates with the increase in temperature, where compared with *c* value at 40 °C, the result at 70 °C decreased by 53.0%. When 0.3% basalt fiber is added into the mixture, the parameters *c* and *φ* of the mixture are improved by 7.9% and 4.6% at 60 °C, respectively. Furthermore, the prediction model of the parameters *c* and *φ* with temperature is put forward, and the prediction models of the shear stress strength and shear stress level on the failure surface with confining pressure are further obtained.
(2) TDCTs indicate that the instability cyclic times *F_N_* of the asphalt mixture decreases exponentially with the increase in the vertical pressure stress levels under the same confining pressure and increases gradually with the increase in confining pressure. Moreover, through analyzing the rules of *F_N_* with shear stress ratio at 60 °C, a uniform instability prediction model for the mixture is obtained to eliminate the effect of different confining pressures. Meanwhile, the reliability of the prediction model is verified by the experimental research and forecast analysis for shear strength parameters of fiber mixture addition.
(3) TDCTs under different temperatures show that the instability life of the asphalt mixture decreases with the increase in temperature, and temperature greatly influences the parameters of the mixture in the instability prediction model. The prediction model parameter values can be calculated using an interpolation method for a given temperature, that is, the instability cyclic times *F_N_* of the same gradation mixture in the entire temperature range can be predicted. Moreover, the reliability of the prediction model in the entire temperature range is further proved by the corresponding TDCT study.

## Figures and Tables

**Table 1:** Shear parameters of SBS asphalt mixture at different temperatures.

Temperature/°C	Vertical Pressure Strength/MPa			*c*/MPa	*φ*/°
	Confining Pressure 0 kPa	Confining Pressure 69 kPa	Confining Pressure 138 kPa		
40	2.291	2.630	2.969	0.5168	41.43
50	1.735	1.978	2.221	0.4623	33.90
60	1.176	1.475	1.774	0.2825	38.68
70	0.975	1.253	1.531	0.2429	37.04

**Table 2:** Shear parameters of basalt fiber asphalt mixture.

Materials	Vertical Pressure Strength/MPa			*c*/MPa	*φ*/°
	Confining Pressure 0 kPa	Confining Pressure 69 kPa	Confining Pressure 138 kPa		
BF	1.321	1.645	1.969	0.3048	40.46

**Table 3:** *F_N_* of fiber asphalt mixture under the 138 kPa confining pressure.

*σ*_0_ (MPa)	*τ*_0_ (MPa)	*σ_n_* (MPa)	*τ_n_* (MPa)	*τ_n_*/*τ*_0_	*F_N_* (times)		
					Group Ⅰ	Group Ⅱ	Group Ⅲ
1.969	0.6966	0.9	0.3763	0.5402	12,060	7820	8710
		1.0	0.4169	0.5984	4620	7610	6110
		1.2	0.4879	0.7004	3030	5020	1910
		1.5	0.5784	0.8302	810	1850	650

## Data Availability

Data sharing is not applicable to this article.
